# Engaging women to set the research agenda for assisted vaginal birth

**DOI:** 10.1111/hex.14054

**Published:** 2024-06-14

**Authors:** Maria R. Torloni, Lucia F. Campos, Arantza Coullaut, Katharina Hartmann, Newton Opiyo, Meghan Bohren, Mercedes Bonet, Ana P. Betrán

**Affiliations:** ^1^ Evidence Based Health Care Post‐graduate Program Sao Paulo Federal University Sao Paulo Brazil; ^2^ Prodigioso Volcán Madrid Spain; ^3^ Mother Hood e.V. Bonn Germany; ^4^ Department of Sexual and Reproductive Health and Research, UNDP/UNFPA/UNICEF/WHO/World Bank Special Programme of Research, Development and Research Training in Human Reproduction (HRP) World Health Organization Geneva Switzerland; ^5^ Gender and Women's Health Unit, School of Population and Global Health, Nossal Institute for Global Health University of Melbourne Melbourne Victoria Australia

**Keywords:** assisted vaginal birth, extraction, obstetrical, patient and public involvement, research design, research priority

## Abstract

**Introduction:**

Public and patient involvement can provide crucial insights to optimise research by enhancing relevance and appropriateness of studies. The World Health Organization (WHO) engaged in an inclusive process to ensure that both technical experts and women had a voice in defining the research gaps and needs to increase or reintroduce the use of assisted vaginal birth (AVB) in settings where this intervention is needed but unavailable or underused.

**Methods:**

We describe the methods and outcomes of online workshops led by WHO to obtain women representatives' perspectives about AVB research gaps and needs.

**Results:**

After technical experts created a list of research questions based on various evidence syntheses, WHO organised four online workshops with 31 women's representatives from 27 mostly low‐ and middle‐income (LMIC) countries. Women rated the importance and priority of the research questions proposed by the technical experts, improving and broadening some of them, added new questions, and voiced their main concerns and views about AVB. Women helped to put the research questions into context in their communities, highlighted neglected factors/dimensions that influence practices and affect women's experience during labour and childbirth, underscored less salient consequences of AVB, and highlighted the main concerns of women about research on AVB. The consolidated vision of technical experts and women's representatives resulted in a technical brief published by WHO. The technical brief is expected to stimulate global research and action closely aligned with women's priorities.

**Conclusions:**

We describe a successful experience of engaging women, mostly from LMICs, in the identification of research gaps and needs to reintroduce AVB use. This process contributed to better aligning research questions with women's views, concerns, and priorities. Given the scarcity of reports about engaging women from LMICs to optimise research, this successful experience can serve as an inspiration for future work.

**Patient or Public Contribution:**

Women representatives were involved at every stage of the workshops described in full in this manuscript.

## 1 INTRODUCTION

High‐quality care during labour and birth could prevent up to 1.3 million intrapartum stillbirths each year, reduce preventable maternal and neonatal mortality, decrease the number of babies with long‐term disability, and improve child development.^1^ Prolonged second stage of labour (when the cervix is fully dilated) complicates 3%–14% of all births and is associated with increased risks for mortality and severe maternal and perinatal morbidity.^2^, ^3^, ^4^, ^5^, ^6^ Currently, the two options to address prolonged second stage are assisted vaginal birth (AVB) and caesarean section (CS).

AVB, also known as instrumental or operative vaginal birth, refers to the use of an instrument such as a forceps or a vacuum extractor, with the objective of achieving a vaginal birth.^7^ AVB is an essential obstetric intervention that can improve outcomes and reduce maternal and perinatal morbidity and mortality.^8^ However, despite the World Health Organization's (WHO) recognition of AVB as one of the seven essential functions of basic emergency obstetric care,^9^ access to AVB is disproportionally low for women in low‐ and middle‐income countries (LMICs). The prevalence of AVB ranges from 4% to 15% in most high‐income countries while it is less than 1% in many LMICs, underlying a persistent global inequity in access to a life‐saving procedure.^10^, ^11^, ^12^, ^13^, ^14^, ^15^, ^16^, ^17^ In addition, over the last decades, even in high‐income settings, there has been a decline in the rate of AVBs that has been accompanied by a steady increase in births by CS.^10^, ^18^, ^19^, ^20^

Potential factors that contribute to the decrease of AVB include lack of trained and experienced health providers capable of indicating and safely conducting an AVB, lack of functioning equipment to perform AVBs, and fear of litigation in case of adverse outcomes.^21^, ^22^ Performing a CS when an AVB would be medically indicated can have serious implications for the health of the mother and newborn, especially in low‐resource settings where women have limited access to comprehensive obstetric care.^23^ Moreover, the risks of a CS performed in the second stage of labour, when the baby's head is deeply engaged in the birth canal, are higher than those of a CS performed in the first stage of labour (before full cervical dilation) or of a prelabour CS.^24^, ^25^, ^26^, ^27^, ^28^ In many cases, a correctly indicated and conducted AVB would be safer and more expeditious than a second‐stage CS.^6^, ^29^ This situation has sparked the interest of many health authorities and clinicians about how to safely increase or reintroduce the use of AVB for women who need this intervention, particularly in LMICs.^30^

Within this context, WHO engaged in an inclusive process to improve understanding of the research gaps and needs to increase access to or reintroduce the use of AVB, especially in LMICs. First, researchers conducted global evidence syntheses of quantitative and qualitative research on barriers and facilitating factors associated with the use of AVB, and the effectiveness of interventions to increase AVB use.^21^, ^22^, ^30^ Then, the WHO convened a technical consultation with a panel of 37 international experts to discuss the research gaps and needs related to AVB and develop of list of research questions that address these gaps and needs. Finally, conscious of the importance of public and patient involvement (PPI) in health research,^31^, ^32^ the WHO convened online group discussions (hereafter designated as ‘workshops’) with women's representatives from different geographical locations to gather their views about the list of research questions compiled by the technical panel, and their concerns about AVB. This manuscript describes the process and results of the PPI workshops conducted with women's representatives to understand their views and perspectives about the list of research questions on AVB that was created by technical experts, and additional research questions proposed by the workshop participants which were deemed relevant to women.

## 2 METHODS

We adapted the methodology of the ACTIVE framework^33^ (designed for describing stakeholder involvement in systematic reviews) for our work. Table 1 depicts the adapted ACTIVE framework as applied to the PPI workshops and the types of engagement for the project. We used a multi‐methods approach to engage with the PPI representatives, via surveys and engagement workshops. We followed the Guidance for Reporting Involvement of Patients and the Public (GRIPP2) reporting guidelines.^34^

**Table 1:** Principles of ACTIVE framework applied.

ACTIVE framework construct	Category
Who was involved?	Women's group representatives
How were stakeholders recruited?	*Mixed open and closed recruitment* WHO offices Lists of women's representatives who had contributed in the past to the development of WHO guidelines on maternal health Collaborators active in the field Snowballing
What was the mode of involvement?	One‐time, direct interaction
At what stage did the involvement occur? What was the level of involvement at each stage?	Develop question: leading, influencingPlan methods: contributing

### 2.1 Aim of the workshops

The overall purpose of involving women's representatives was to improve the quality and relevance of future research on AVB, increase transparency, and gain public trust.

The specific aim of the workshops was to obtain women's views on the importance and priority of the research questions about AVB identified by a group of technical experts convened by WHO^35^ (Annex S1), their comments about the questions, additional research questions relevant to women, and hear their concerns about AVB.

### 2.2 Participants

This purposive sample of participants consisted of representatives of support and advocacy groups of women worldwide. It was not required that the participants had experienced operative births themselves, neither was it necessary that the participants be health care professionals. Special efforts were made to include women from LMICs. WHO strived to include women, groups or organisations that actively work with and are led by women in all their diversity, in the fields of sexual or reproductive health, women and childbirth rights, obstetric violence and/or gynaecologist and obstetrics. Participants were identified via WHO offices, lists of women's representatives who had contributed in the past to the development of WHO guidelines on maternal health (to provide women's perspectives to guideline development), collaborators active in the field, and snowballing technique. Once identified, groups or women were asked for assistance in identifying other potentially relevant participants. When organisations were contacted, they proposed a specific woman within the organisation best suited to participate in the workshop.

### 2.3 Preworkshop survey

One week before the scheduled workshops, each participant was asked to complete an anonymous online survey (Annex S2) to rate the importance and priority of the list of research questions that was created by the 37 technical experts convened by WHO at a previous meeting. These questions were grouped into four major categories: (i) Women's and Communities' views, (ii) Training and Clinical aspects, (iii) Implementation, and (iv) Sustainability. Five of the original 31 research questions proposed by the technical experts were excluded because they required specific previous knowledge or background unfamiliar to most women's representatives (i.e., clinical guidelines, operational implementation research, the WHO Labour Care Guide, and the role of professional associations). Participants were asked to rate each research question on a Likert scale as to its degree of importance (1 = *not important*, 5 = *very important*) and priority (1 = *very low*, 5 = *very high priority*). Importance was defined as the interest, significance, and value of the research question for women. Priority referred to the degree to which the question needed to be prioritised or promptly addressed as a research topic. We present the results of the survey descriptively (number and percentages). The survey was not the main outcome of this process, but it helped prepare the women for the workshops. It was not mandatory and women were invited to complete and submit it as they wished.

### 2.4 Workshop format

Women's representatives were grouped in four online workshops with similar linguistic and geographical backgrounds. Two workshops were conducted in English (at different times due to the geographic location of the participants), and one each in French and Spanish. Each workshop lasted 1.5 h and was facilitated by two independent professional facilitators (AC and LFC). Transparent reflexivity throughout qualitative research is central to good practice. AC is a journalist and LFC is a sociocultural anthropologist. Neither have clinical experience nor have conducted research on AVB or maternity care. They both believe pregnant women should have full access to information and knowledge of all matters regarding their pregnancy, labour, and childbirth. They both understood and encouraged the need for a safe space for participants to freely express their opinions. The interdisciplinary research team considered its potential biases before and throughout the workshops, and in summarising the discussions of the workshops to acknowledge how their perspectives of AVB may influence the analysis and presentation of findings.

At the beginning of each workshop, WHO staff (APB) introduced the facilitators to the group and stayed throughout the workshop but did not participate in the discussion unless she was called upon to clarify something. The facilitators asked the participants to introduce themselves and shared on the screen the list of research questions suggested by the group of technical experts for future research to optimise the use of AVB. The facilitators then encouraged participants to comment on and give their views about the importance and priority, or other perspectives, about each of the research questions or major categories, using the following guiding questions:

1. How important and urgent are these research questions and topics to women who give birth in the communities/settings/countries that you represent?
2. What important research questions that need to be addressed in future studies are missing from the list?
3. What are the main areas of concern or worries that women have regarding AVB?

Workshops were conducted in Zoom and video recorded to facilitate qualitative content analysis. All participants gave oral informed consent for these recordings, the anonymous transcription and analyses of their statements and answers to the pre‐workshop survey, and use of this information by WHO. Participants were informed that everything shared and discussed during the workshops would be summarised and would inform the writing of documents prepared by WHO on AVB, including the writing of a scientific manuscript, and future research activities. We used a content analysis approach to explore the identify and quantify patterns in communication from the workshops, interpret the meanings behind the participant contributions, and the relationships between different ideas and concepts discussed.^36^ LCF and AC led the content analysis, and LFC, AC, and APB led the survey analysis. All results were discussed and further interpreted among co‐authors to improve trustworthiness of the analysis.

## 3 RESULTS

**Figure 1:**
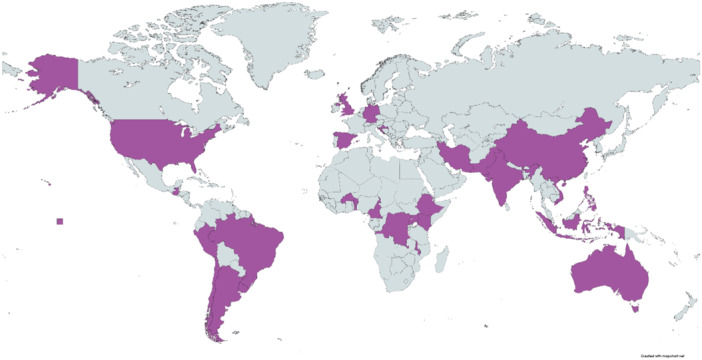
Geographic representation of women participating in the workshops.

Between April and May 2022, four virtual workshops, including 31 representatives of women and advocacy groups from 27 countries were held to seek their views on the research questions on AVB proposed by technical experts. Two workshops were conducted in English (12 and 8 participants), one in French (four participants), and one in Spanish (seven participants). Most of the participants were from middle‐ (*n* = 16) and low‐income countries (*n* = 6) (Figure 1 and Annex S3).

### 3.1 Importance and priority of the research questions

**Figure 2:**
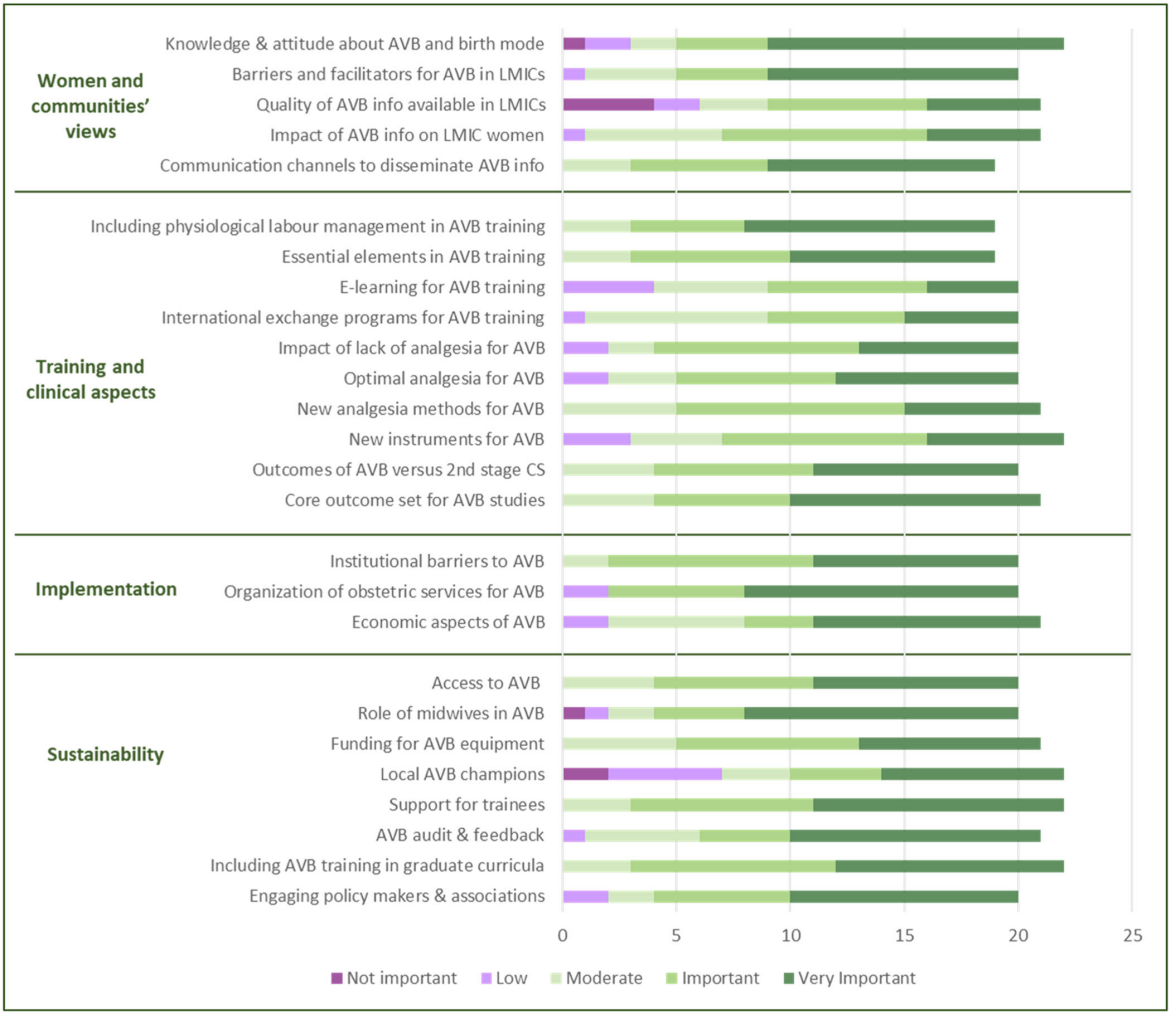
Importance of research questions about assisted vaginal birth (AVB). Importance: interest, significance, and value of questions for women. The graph shows the number of respondents who selected each option for each specific research question. The questions are abbreviated; see complete questions in Annex S1.

**Figure 3:**
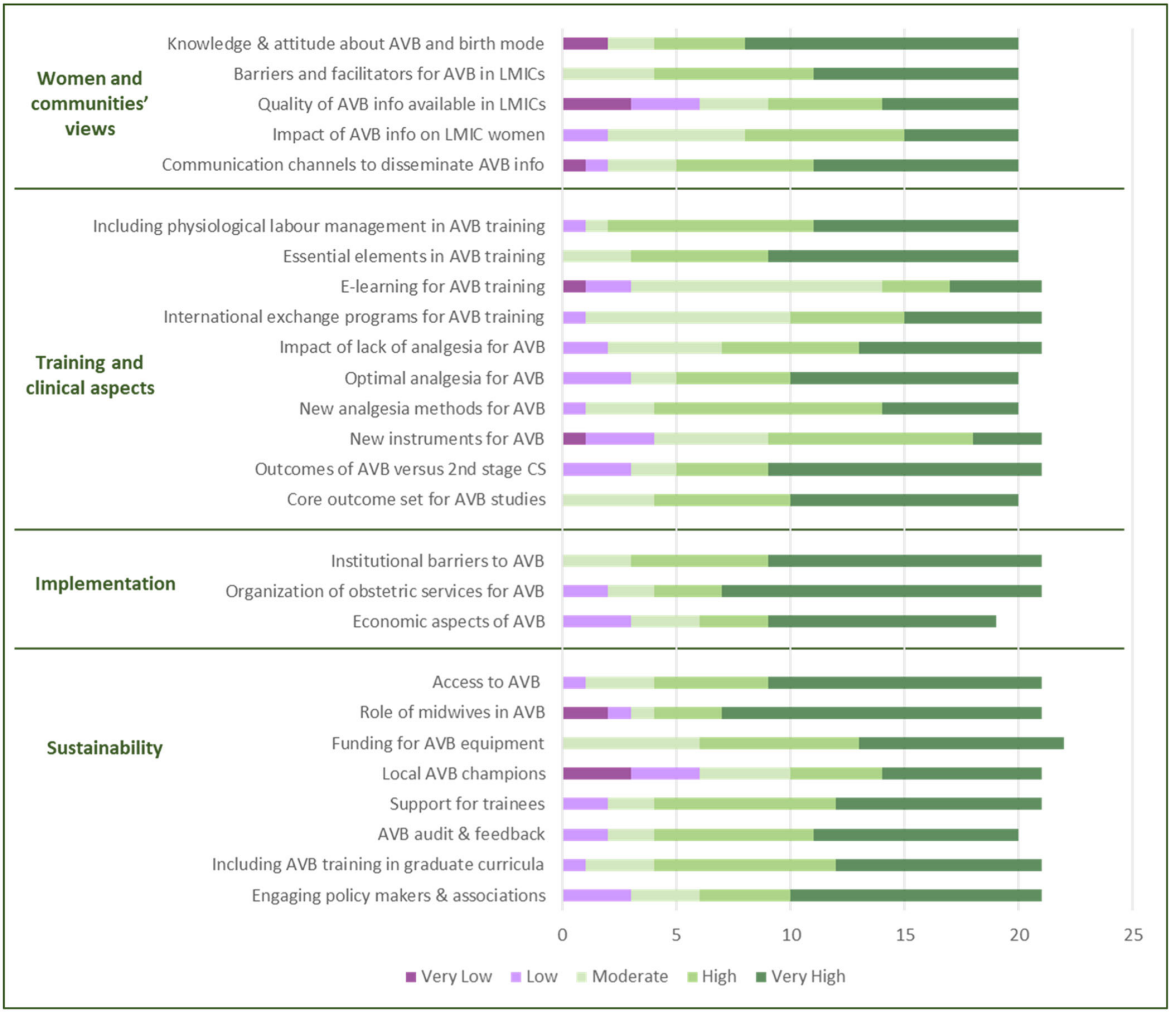
Priority of research questions about assisted vaginal birth (AVB). Priority: need for prompt action, need to be considered a priority research topic, for women. The graph shows the number of respondents who selected each option for each specific research question. The questions are abbreviated; see complete questions in Annex S1.

Overall, 22 of the 31 participants answered the anonymous survey, and some of them did not answer all questions. Figures 2 and 3 present participants' assessment of the importance and priority of the research questions proposed by the technical panel. There was congruence between scores for importance and priority in all domains. Specific ratings of participants from different settings are presented in Annex S4. The research questions on *‘Women's and Communities' views’* rated as the most important were those about women's knowledge/attitude about AVB, barriers for women to access AVB in LMICs, and the best communication channels to disseminate information about AVB; these same questions also received the highest scores in terms of priority. In the ‘*Training and Clinical aspects*’ category, participants rated questions about including training on physiology of labour in AVB courses and the creation of core outcomes for AVB studies as being the most important. In this same category, high‐quality studies comparing outcomes of AVB versus second‐stage CS, and studies to identify the essential elements for effective AVB training received the highest priority ratings. In the ‘*Implementation*’ category, the research question about organisation of local maternity services to ensure timely access to high‐quality AVB for all women received the highest importance and priority ratings. In the ‘*Sustainability*’ category, the question on how to explore and expand the role of midwives in performing AVB received the highest importance and priority ratings.

### 3.2 Workshop discussions: Women's views on major areas of AVB research

Table 2 and Annex S5 present participants' comments about the questions in the four major areas of research on AVB proposed by the panel of experts. There were no major differences in the perspectives among the four workshop groups.

**Table 2:** Views and comments of women's representatives about areas of research on AVB.

Original research questions from technical experts^a^	Women's views and comments	Expansion of or additional research questions
Women's and communities' issues		
1. Knowledge and attitude about AVB and birth mode. 2. Barriers and facilitators for AVB in LMICs. 3. Quality of AVB info available in LMICs. 4. Impact of AVB info on LMIC women. 5. Communication channels to disseminate AVB info.	Safety concerns (fears of damage to baby and maternal physical and emotional health) and how best to inform women about this (4)^b^ Women lack knowledge that AVB is an option when VB is not possible. This lack of knowledge leads them to see CS as the only alternative. This must be addressed (3) It is key to understand how AVB is framed by women, and how and where women obtain, process and are influenced by information about AVB (3) Research is needed on how to change the views of some communities/societies where birth is seen as an illness instead of a physiological process (3) Education is key to avoid misconceptions and harmful beliefs and for decision‐making (2) Research should assess the influence of communities on women's views about AVB (2) It is important not only to assess women's and communities' views and opinions about AVB but also to study how much the opinions/views of women and communities about AVB are taken into consideration by researchers, policymakers and other relevant actors (1) Investigate how to use sensitising strategies to involve policymakers and HCP to invest efforts into providing information about AVB to the general population (1) Research on the sources of influence and the role of decision‐aids (1) It will be challenging to get women's views about AVB in settings where this intervention is almost inexistent (1)	Studies on how to improve women's trust in health care providers and health systems who perform or offer AVB. How to improve the relationship (increase trust, reduce fear) between women and HCP in settings where poor communication between these actors prevails? What is the emotional and symbolic impact of having AVB in specific populations where stigma can be associated with it (e.g., Indigenous women)? Are partners' or husbands' opinions considered? Would these help the woman in making an informed decision? What is the importance and role of support networks or groups for women who have gone through or need to have an AVB? How to involve young people in the efforts to increase VBs in general (motivation, role, innovation)?

AVB training and clinical aspects		
6. Including physiological labour management in AVB training. 7. Essential elements in AVB training. 8. E‐learning for AVB training. 9. International exchange programs for AVB training. 10. Impact of lack of analgesia for AVB. 11. Optimal analgesia for AVB. 12. New analgesia methods for AVB. 13. New instruments for AVB. 15. Outcomes of AVB versus 2nd stage CS. 16. Core outcome set for AVB studies.	It is important to train HCPs about nontechnical skills, such as communication skills and how to treat women with empathy and respect and how to see women not as objects but as people capable of participating in decision making (4) Research on how to train and sustain skills and knowledge at point of care is fundamental (4) It is important to teach/train HCPs (including midwives) to view labour and childbirth as healthy physiological processes that should be respected and how to best support women physically and emotionally during these processes (4) In remote settings (especially in Africa) it is important to investigate how to promote on‐line training, and the exchange of clinical information between HCPs (physicians and midwives), including how to overcome technological barriers in some countries (2) How to motivate and train HCPs who do not want to learn how to do AVBs (1) Explore possibility of team training models facilitated by midwives so that the role of the midwife is integrated into the clinician's practice as well (1)	How to improve the knowledge and skills of midwives in assisting vaginal births in general (not only AVB)? What are the long‐term physical and mental (anxiety, posttraumatic stress disorder, depression) adverse outcomes of AVB and how to best treat them? What is involved in informed consent for AVB and how is it obtained? How often do women participate in decision‐making during their labour and birth? Are values and preferences of the woman/her community about AVB considered? How to ensure that AVB will only be done when and if needed, and with the consent of women? What are the consequences of unnecessary AVB and disrespect during AVB?

Implementation of AVB		
17. Institutional barriers to AVB. 18. Organisation of obstetric services for AVB. 22. Economic aspects of AVB	Financial resources and the supply of instruments are essential for implementation (3) Active engagement and participation of government and policymakers committed to improving quality of care for labour and delivery in general, including AVB, is essential for change (3) How to overcome the knowing‐doing gap (i.e., how to overcome barriers to implement what is known to work) (2) How to manage political and financial barriers, and insurance limitations (if CS are more profitable for the system, it will be difficult to change) (2) It will be necessary to renovate buildings and delivery rooms to offer AVB (clinical practice depends on structural changes) (2) Health systems are perceived to meet the needs of providers before those of women. Strategies need to address the concerns of both (1) Human rights must be ensured during implementation (1) How to ensure that companionship is allowed in the moment that the women is most vulnerable (1) Particularly in settings with limited resources, sanitation and hygiene need to be ensured (1)	What is the cost of AVB and how does it act as a potential barrier to AVB? How to ensure the presence of a birth companion for all women who undergo AVB?

Sustainability of AVB		
23. Access to AVB. 24. Role of midwives in AVB. 25. Funding for AVB equipment. 26. Local AVB champions. 27. Support for trainees. 28. AVB audit and feedback. 30. Including AVB training in graduate curricula. 31. Engaging policymakers and associations	Continuous flow of information must be provided to all actors involved: women, clinicians, midwives (4) How to operationalise task‐shifting where appropriate and possible: it is important that midwives be allowed to do AVB to ensure the sustainability of AVB, especially in rural and remote areas (3) Midwives need to be given more power, skill sets and knowledge. Their competences and abilities must be recognised (2) In some countries, ‘AVB champions’ may have a negative impact because associated incentives to perform AVB may disregard and run over women's opinions and rights. Therefore, it is important to investigate possible negative effects of ‘champions’ on AVB use/acceptability (1) Clear guidelines on indications for AVB and CS are needed (1)	What are the roles and responsibilities of midwives regarding AVB in different settings?

Abbreviations: AVB, assisted vaginal birth; CS, caesarean section; HCP, health care provider; LMIC, low‐ and middle‐income countries; VB, vaginal birth.

^a^ The questions are abbreviated; see full questions in Annex S1.

^b^ Number of workshops where the topic or issue was discussed.

#### 3.2.1 Research questions related to women's and communities' views

Participants from all four workshops agreed that research must address the safety concerns associated with AVB, and emphasised the importance of research on how to best inform women on this topic. Participants from most geographic regions also commented that research should focus on women's lack of knowledge about AVB as an alternative to CS, misconceptions about AVB, how women can obtain reliable information about AVB, and how to change the views of communities or societies towards pregnancy and childbirth as physiological instead of pathological processes.

#### 3.2.2 Research questions related to training and clinical aspects

Participants from the four workshops emphasised the crucial importance of well‐trained, skilled and competent professionals, and agreed on the need for research on how to train health care providers (HCPs) about nontechnical skills alongside AVB skills. Soft or nontechnical skills include cognitive (e.g., situational awareness) and social skills (e.g., communication and teamwork).^37^ Additionally, participants in all workshops stressed the importance of research about how to sustain knowledge and skills at the point of care, and how to train HCPs to view and manage labour and childbirth as physiological processes that need to be supported, rather than pathological processes that require interventions. Participants in two of the workshops also commented on the need for research about the feasibility and effectiveness of remote e‐training for AVB, particularly in Africa.

#### 3.2.3 Research questions related to implementation

In three of the workshops, women's representatives emphasised the need for research and innovative approaches on how to ensure the financial resources needed to implement AVB (instruments, staff training) and how to engage members of governments and policymakers to foster and promote respectful and high‐quality maternity care. Studies on how to improve health care systems and overcome related financial barriers to implement AVB were also mentioned as necessary by participants in two of the workshops.

#### 3.2.4 Research questions related to sustainability

Participants in all four workshops agreed that it was important to conduct research on how to ensure a constant, clear, and consistent flow of information between women and HCPs to ensure better decision‐making and respect. Participants also pointed to the need for research on task‐sharing so that midwives could undertake AVB where appropriate and feasible, and how to improve their competencies to ensure the sustainability of AVB, especially in remote or less resourced areas.

### 3.3 Expansion of and additional research questions proposed by women

The participants expanded several of the research questions proposed by the technical experts related to the four main themes. They also proposed several additional research questions related to Women's and Communities views about AVB, and Training and Clinical aspects (Table 2).

### 3.4 Women's concerns about AVB

The participants listed 12 issues as being the major concerns of women about AVB (Table 3 and Annex S6). Safety of AVB for the baby was mentioned as a major concern in all workshops, and safety of AVB for women was mentioned in three workshops. Other concerns mentioned in three out of the four workshops were the poor communication between HCPs and women who need AVB, the pressure put on women to sign informed consent for AVB without adequate information, the lack of information leading women to fear AVB, and women's perception that HCPs do not respect the physiology of labour and their possible lack of skills to perform AVB.

**Table 3:** Main concerns of women about AVB.

Concerns	Number of workshops^a^
Safety
Safety of AVB for baby	4
Safety of AVB for women (physical and emotional/mental health)	3
Unavailability of treatment, follow‐up and support in case of adverse maternal or neonatal outcomes	2
**Healthcare providers skills and health systems**	
Fear that HCPs are not competent/skilled to do AVB	3
Disrespect of HCPs for physiology of labour and birth, nonhumanised care leading to maternal trauma	3
Maternal risks due to lack of hygiene of instruments and rooms where AVB occur	1
The fear that women have of midwives	1
Excess use of unnecessary AVBs as part of the obstetric violence scenario or effort to decrease CS rates	1
Social inequality in access to good quality health care by most socially disadvantaged or poorest pregnant women	1
**Communication and information**	
Poor communication between HCPs and women	3
Pressure on women to sign AVB informed consent without adequate information	3
Women's insufficient information about AVB which contributes to fear	3

Abbreviations: AVB, assisted vaginal birth; CS, Caesarean section; HCP, health care provider.

^a^ Number workshops where this was mentioned.

### 3.5 Use of women's contributions

The three‐step process (evidence syntheses, consultation with technical experts, and workshops with women's representatives) informed the development of a WHO technical brief released in 2023.^35^ The technical brief documents the process and lists the original research gaps and needs proposed by the technical experts, women's views about and specific contributions to some of these questions and the additional research questions that they proposed to address women's concerns. The technical brief aims to raise awareness about the need to generate crucial evidence to advance this topic and stimulate action to reintroduce AVB as an alternative to CS, in cases when this intervention is appropriate.

## 4 DISCUSSION

This manuscript presents in detail the views, concerns, and suggestions of 31 women's representatives from 27 different countries about research questions to reintroduce AVB use initially proposed by technical experts. In general, women agreed about the importance of the research questions proposed by the technical experts but added enriching nuances, emphasising aspects and dimensions of special importance to them.

Safety of AVB for babies and women stood out as a major concern for women. Therefore, the importance of research on how to most efficiently train HCPs, and how to maintain their skills and competence to safely conduct AVB cannot be overstated and remains a challenge in contemporary emergency obstetrics.^38^, ^39^ However, training also needs to integrate additional dimensions that are crucial for women such as ensuring that HCPs regard labour and childbirth as physiological processes that need to be supported, instead of pathological conditions that always demand interventions, which aligns with recent WHO intrapartum recommendations for a positive childbirth experience.^40^ Similarly, women highlighted the need for research that recognises the importance of and incorporates nontechnical skills, including cognitive and social skills. Not only ‘what it is done’ but ‘how it is done’ is valued by women.

Women endorsed the importance of research about women's and communities' views on AVB, reflecting the relevant role of societal influence on birth preferences.^41^, ^42^, ^43^ Participants emphasised the need to study what is the current knowledge of women and communities in LMICs about mode of birth in general and about AVB, as well as their attitudes towards both, since these are the starting point for change. Women underscored the importance of identifying the most efficient ways to inform women in contemporary societies, including through the use of mass and social media channels. Women's representatives also emphasised the importance of helping women to find reliable information and avoid misinformation about AVB in modern communication channels, especially in social media, where unreliable information is ubiquitous and easily accessible.^44^, ^45^, ^46^, ^47^

Most organisations view PPI in health research, including prioritisation exercises, planning, design or conduct of studies and dissemination of results, as an important factor to improve the quality and relevance of research.^31^, ^32^, ^48^, ^49^ PPI ensures that research focuses on aspects and outcomes that are relevant and matter to the patients, and enhances ownership and responsiveness to results and recommendations.^31^, ^32^, ^50^ Our experience confirms the importance of including women's representatives in the early stages of the research process. Women's views at this stage helped to improve, complement, and broaden research questions formulated by technical experts. Besides proposing additional questions relevant to women in different settings, women's representatives helped to put into context the research questions proposed by the technical experts. The contributions that emerged from the workshops with women highlighted more silent factors or dimensions that influence practices and affect women, illustrate the complexity of the milieu that women experience during labour and childbirth, and underscore less salient consequences. These nuances were reflected in the subsequently published WHO technical brief,^35^ proposing areas and questions where research are most needed to reintroduce AVB.

This was our first experience organising virtual workshops to expand the contribution of women to research beyond the typical WHO approach of including one or two women's representatives in technical meetings. The workshops were carefully planned and organised to foster focus and maximise participants' time, while simultaneously cultivating an atmosphere of trust and respect, free of judgement, where all opinions were welcomed and had a space. Women engaged easily and respectfully, and were eager to contribute and share their views and the experiences of the women whom they represented. Language barriers were largely reduced by conducting workshops in three languages (English, French and Spanish).

There are few reported experiences from LMICs on the use of methodological tools and guidelines for PPI in research.^51^, ^52^, ^53^ This manuscript documents a successful and positive experience using virtual on‐line workshops for this purpose. If adequately organised, this can be an affordable approach to obtain the views and perspectives of women from a large number of countries, avoiding tokenism. Digital events and discussions are becoming a popular and less expensive alternative to in‐person interactions to exchange information with and from patients, experts and researchers.^54^, ^55^ However, poorly prepared digital events may result in participant disengagement and disinterest. Participants need to have a positive experience, feel satisfied, and know that their time and contribution are respected.^56^ We found that sharing the research questions in advance with the participants and conducting a pre‐workshop survey allowed women to arrive more prepared to the workshops, facilitating readiness and effectiveness. We acknowledge that the fact that one‐third of participants did not answer the survey could have affected the validity of its results. However, the survey was not the main focus of this process but a way of helping the women prepare for the workshops. To ensure inclusive participation and optimal results, it is important to schedule workshops at times suitable for different geographic settings, conduct meetings in different languages, and ensure reliable internet connection. Event organisers must invest time and effort in careful planning before these meetings, not only to maximise expected immediate outcomes, but also to motivate individuals to participate in future similar events. Given the scarcity of reports about engaging women from LMICs in research, this successful experience can serve as an example for future work.

Technical and clinical aspects have driven maternal and perinatal health research for decades and are acknowledged by women to be important. However, these can no longer stand alone in the research agenda. It is important to hear women's voice about the need for respectful care, information, and communication as essential ingredients for informed consent and shared decisions.^40^ It is also important to consider how to promote and achieve respectful care in constrained health systems, particularly in LMICs where overburdened health care providers often work in settings that lack infrastructure.^57^

Interventions to improve maternal health and reduce maternal and perinatal mortality and morbidity are increasingly more multidisciplinary, complex, and challenging. If AVB is to be revitalised to improve maternal and perinatal health, research must reconcile clinical and nonclinical aspects. The discussions during the workshops with women underscored that studies on how to improve HCPs' technical skills to conduct AVB must go hand in hand with studies on how to train HCPs to address women's needs for respectful care during childbirth, appropriate communication, reliable information, and shared decision‐making.

## 5 CONCLUSION

We describe a successful experience of engaging women, mostly from LMICs, in the identification of research gaps and needs to reintroduce AVB use. This process contributed to better aligning research questions with women's views, concerns, and priorities. Women agreed with most research questions and topics proposed by the technical experts but added important nuances and dimensions to several questions, processes, and outcomes. Participants also proposed additional research questions, and highlighted the main concerns of women which need to be addressed when planning studies to reintroduce or increase the use of AVB. The consolidated vision of technical experts and women's representatives resulted in a WHO technical brief proposing areas and questions where research is most needed to increase and reintroduce AVB, especially in LMICs. The technical brief is expected to stimulate global research and action closely aligned with women's priorities.

## AUTHOR CONTRIBUTIONS

**Maria R. Torloni**: Methodology; writing—original draft; formal analysis; data curation; investigation; conceptualisation. **Lucia F. Campos**: Writing—review and editing; formal analysis; methodology; data curation. **Arantza Coullaut**: Methodology; writing—review and editing; formal analysis; data curation. **Katharina Hartmann**: Writing—review and editing; formal analysis; validation. **Newton Opiyo**: Validation; writing—review and editing; formal analysis. **Meghan Bohren**: Validation; writing—review and editing; formal analysis; methodology. **Mercedes Bonet**: Writing—review and editing; methodology; formal analysis; funding acquisition. **Ana P. Betrán**: Conceptualisation; funding acquisition; methodology; writing—original draft; formal analysis; supervision.

## CONFLICT OF INTEREST STATEMENT

The authors declare no conflict of interest.

## Supporting information

Annex 1. Summary of research gaps and needs.

Annex 2: Pre‐workshop survey.

Annex 3: Details of participants.

Annex 4: Importance and priority of research questions per geographic region.

Annex 5. Views and comments on research categories per geographic region.

Annex 6. Concerns of women about AVB per geographic region.

## Data Availability

The data that supports the findings of this study are available in the Supporting Information of this article.
