# Supplementary material for: Engaging women to set the research agenda for assisted vaginal birth
Source: Health Expect. 2024 Jun 14;27(3):e14054. doi: 10.1111/hex.14054 (PMC11178515; doi:10.1111/hex.14054)
Supplement: Supplementary file 1 — Annex 1. Summary of research gaps and needs. [file HEX-27-e14054-s001.docx]

**Annex 1. Summary of the research gaps and needs to re-introduce or increase AVB according to panel**

**of technical experts.**

| Theme | Summary of identified gaps and research needs |
| --- | --- |
| Women and communities’ views | 1. What knowledge and attitudes exist among women and communities in LMIC about mode of birth in general and AVB in particular? 2. What are the barriers and facilitating factors for AVB emerging from women and communities from LMIC? What are the behavioral and cultural barriers? 3. What is the quality and comprehensiveness of the information about AVB available in LMIC mass and social media channels? 4. How does the information available in mass and social media channels impact women’s and communities’ knowledge and attitudes towards AVB in LMIC? 5. What are the most effective ways to communicate and disseminate information on AVB to communities in general (e.g., mass media, social media)? What are the best communication strategies in settings where illiteracy is widespread (e.g., dance, songs, videos)? What communication methods can be used in settings where access to internet and social media is limited? |
| Training and clinical aspects | **TRAINING AND SUPPORT**   1. What is the impact of including information and training on maternal and fetal physiology and mechanisms of labor/birth in AVB training courses on AVB use and outcomes? 2. What are the essential elements for effective AVB training? What are the essential elements for effective support and supervision? Is it different in different contexts? 3. Are remote e-learning and mobile technologies acceptable and effective to gain and maintain expertise and to increase AVB use? 4. Explore the potential role of a structured international training exchange program as a platform to facilitate and accelerate AVB skills between different countries/regions by capitalizing on existing knowledge and skills. Note that such an exchange will only be useful if the structure in the receiving setting is in place to allow for AVB.   **MEDICAL TECHNOLOGY**   1. What is the impact of lack of analgesia on AVB outcomes and views? 2. What are the optimal analgesia methods for AVB? What is the acceptability, outcomes, and resource use associated with the optimal method? 3. Discovery research: develop and test better and simpler local analgesia methods for AVB that are instrument and setting appropriate. 4. Discovery research: identify new ways/instruments to conduct AVB.   **POLICY AND MONITORING**   1. Compare and assess the quality of existing AVB guidelines. Produce and disseminate updated and evidence-based AVB guidelines endorsed by major international institutions. 2. High quality studies comparing outcomes of AVB versus second stage caesarean section to better inform healthcare professionals, policy makers and women. 3. Develop a core outcome set for AVB studies including short- and long-term (5 years or later) maternal and neonatal outcomes that matter to relevant stakeholders (including women) and how to measure these outcomes. |
| Implementation | 1. What are the barriers and facilitating factors that influence health systems, organization and culture, supply and human resources training that affect the introduction of AVB techniques within specific health systems in LMIC? 2. How to organize local maternity services so that every woman who needs it has timely access to a high quality AVB, including pain relief and support? 3. Operational implementation research using a systems thinking approach with its toolbox of motivation changers (e.g. accountability, peer pressure, champions, emerging leadership)^1^ 4. How to incorporate the use of AVB within the WHO Labour Care Guide^2^ implementation? What are the effects of this integration on clinical outcomes? 5. Explore the role of professional associations in supporting evidence-based models of care and improved regulation and training in countries 6. Economic evaluations for policy-makers and organizations. |
| Sustainability | 1. Place of care and pathways for AVB; how to facilitate AVB in primary health care settings. 2. Explore and expand the role of midwives: midwife-led models, or midwife-led birth centers embedded within hospitals which provide comprehensive emergency obstetric and newborn care. Note that regulatory and legal restrictions for midwives and necessary changes are important to consider within the local contexts. Research to assess the feasibility, acceptability, and impact of these models on clinical outcomes is necessary. 3. How to ensure funding and appropriate mechanisms to acquire, replace, and maintain essential equipment including instruments needed for AVB. 4. What is the impact of Champions ^3^ ? How to make use of local Champions. If no local Champions are available, how to support international experts in a sustainable and effective manner? What do Champions need? 5. How to ensure appropriate ongoing support to trainees, especially when re-introducing AVB. 6. Explore the impact of regular local/regional audit and feedback (on AVB rates and core maternal and perinatal outcomes) on sustainability of AVB use. 7. Explore the impact of creating, adapting and disseminating national guidelines on AVB. 8. Explore the long-term impact of including AVB training in the formal curricula of medical schools, residency programs, nursing, and midwifery schools and in-service education programs. 9. How to use advocacy strategies and engage policy makers and professional associations more effectively? How to engage policy makers to encourage evidence-based practices more effectively? |

1. Adam T, de Savigny D. Systems thinking for strengthening health systems in LMICs: need for a paradigm shift. Health Policy Plan. 2012;27 Suppl 4:iv1-3.

2. WHO labour care guide: user’s manual. Geneva: World Health Organization; 2020.

3. Although there is no standard definition of a champion in the implementation literature, common elements of a champion for supporting change in healthcare settings include being a staff member (who either volunteers or is assigned an additional level of responsibility), who may perform a number of different roles in order to improve staff adherence to a particular guideline, policy or intervention ( Hall AM, Flodgren GM, Richmond HL, Welsh S, Thompson JY, Furlong BM, et al. Champions for improved adherence to guidelines in long-term care homes: a systematic review. Implement Sci Commun. 2021;2(1):85
