# Supplementary material for: Engaging women to set the research agenda for assisted vaginal birth
Source: Health Expect. 2024 Jun 14;27(3):e14054. doi: 10.1111/hex.14054 (PMC11178515; doi:10.1111/hex.14054)
Supplement: Supplementary file 2 — Annex 2: Pre‐workshop survey. [file HEX-27-e14054-s005.docx]

**Annex 2: Pre-workshop questionnaire**

Questionnaire for women’s views [Monkey survey]. English version

Thank you for agreeing to participate in the consultation (video-conference) to provide your views and opinions on research questions to optimize the use of assisted vaginal birth (AVB), especially in low- and middle-income countries. These research questions were created by a group of experts (including doctors, midwives, policy-makers, and researchers) in a meeting convened by the World Health Organization in December 2021 and now we need to hear from you before we can move forward.

Before the video-conference, we are asking you to please rate the proposed research questions according to two criteria: **importance** and **priority**:

- **Importance:** interest, significance, and value for women.
- **Priority:** need for prompt action, need to be considered a priority research topic.

Below you will find the list of the research questions. Please, apply these two criteria to rate each of the questions. Rating goes from 1 to 5: 1 is the lowest rating (i.e., not important; not a priority) and 5 represents the highest rating (i.e., very important; high priority). We will combine the ratings of all respondents and list the most highly rated topics by each of the two criteria. We will share this information with you during the video-conference (workshop). The rating process should take approximately 10 minutes. Many thanks for your time.

|  |  |  |  |  |
| --- | --- | --- | --- | --- |
| Research questions/topics | | | **Importance**  (1-5)  1=not important 5=very important | **Priority**  (1-5)  1=not a priority  5=high priority |
| WOMEN AND COMMUNITIES’ VIEWS | | | | |
| What knowledge and attitudes exist among women and communities in low and middle income countries (LMIC) about mode of birth in general and assisted vaginal birth (AVB) in particular? | | |  |  |
| What are the barriers and facilitating factors for AVB emerging from women and communities from LMIC? What are the behavioral and cultural barriers? | | |  |  |
| What is the quality and comprehensiveness of the information about AVB available in LMIC mass and social media channels? | | |  |  |
| How does the information available in mass and social media channels impact women’s and communities’ knowledge and attitudes towards AVB in LMIC? | | |  |  |
| Which channels (TV, social media, radio…) are most effective to inform on AVB? In case of illiteracy, what kind of format (dance, songs, videos…) would be most effective? What communication methods can be used in settings where access to internet and social media is limited? | | |  |  |
| TRAINING AND CLINICAL ASPECTS | | | | |
| *Training and support* | | |  |  |
| What is the impact of including information and training on maternal and fetal physiology and mechanisms of labor/birth in AVB training courses on AVB use and outcomes? | | |  |  |
| What are the essential elements for effective AVB training? What are the essential elements for effective support and supervision? Does it change in different contexts? | | |  |  |
| Are remote e-learning and mobile technologies acceptable and effective methods to gain and maintain expertise, and to increase AVB use? | | |  |  |
| Would a structured international training exchange program be helpful in facilitating and accelerating AVB skills between countries/regions? (Would only be useful if the structure in the receiving setting is in place to allow for AVB) | | |  |  |
| *Medical technology* | | |  |  |
| What is the impact of lack of analgesia on AVB outcomes and views? | | |  |  |
| What are the optimal analgesia methods for AVB? What is the acceptability, outcomes, and resource use associated with the optimal method? | | |  |  |
| Discovery research: develop and test better and simpler local analgesia methods for AVB that are instrument and setting appropriate. | | |  |  |
| Discovery research: identify new ways/instruments to conduct AVB. | | |  |  |

| Policy and monitoring |  |  |
| --- | --- | --- |
| High quality studies comparing outcomes of AVB versus second stage caesarean section so that healthcare professionals, policy makers and women are better informed. |  |  |
| Develop a core outcome set for AVB studies including short- and long-term (5 years or later) maternal and neonatal outcomes that matter to relevant stakeholders (including women) and how to measure these outcomes. |  |  |
| IMPLEMENTATION | | |
| What are the organizational, cultural, supply and human resources barriers that affect the introduction of AVB techniques within specific health systems in LMICs? What factors facilitate it? |  |  |
| Information and methods on how to organize local maternity services so that every woman who needs it has timely access to a high quality AVB, including pain relief and support. |  |  |
| Economic evaluations for policy-makers and organizations. |  |  |
| SUSTAINABILITY | | |
| Where to access AVB? How to facilitate/support AVB in primary health care settings? |  |  |
| Explore and expand the role of midwives. For example: midwife-led models, or midwife-led birth centers embedded within hospitals which provide comprehensive emergency obstetric and newborn care. Research to assess the feasibility, acceptability, and impact of these models on AVB outcomes and outcomes in general. (Depending on regulatory and legal restriction) . |  |  |
| How to ensure funding and appropriate mechanisms to acquire, replace, and maintain essential equipment including instruments needed for AVB. |  |  |
| What is the impact of Champions*? How to make use of local Champions. If no local Champions are available, how to support international experts in a sustainable and effective manner? What do Champions need? |  |  |
| How to ensure appropriate ongoing support to trainees, especially when re-introducing AVB. |  |  |
| Explore the impact of regular local/regional audit and feedback (on AVB rates and core maternal and perinatal outcomes) on sustainability of AVB use. |  |  |
| Explore the long-term impact of including AVB training in the formal curricula of medical schools, residency programs, nursing, and midwifery schools and in-service education programs. |  |  |
| How to engage policy makers and professional associations more effectively? What strategies are better? How to engage policy makers to encourage evidence-based practices more effectively? |  |  |

* Although there is no standard definition of a champion in the implementation literature, common elements of a champion for supporting change in healthcare settings include being a staff member (who either volunteers or is assigned an additional level of responsibility), who may perform a number of different roles in order to improve staff adherence to a particular guideline, policy or intervention (Hall AM, Flodgren GM, Richmond HL, Welsh S, Thompson JY, Furlong BM, et al. Champions for improved adherence to guidelines in long-term care homes: a systematic review. Implement Sci Commun. 2021;2(1):85)
