# Supplementary material for: Engaging women to set the research agenda for assisted vaginal birth
Source: Health Expect. 2024 Jun 14;27(3):e14054. doi: 10.1111/hex.14054 (PMC11178515; doi:10.1111/hex.14054)
Supplement: Supplementary file 3 — Annex 3: Details of participants. [file HEX-27-e14054-s006.docx]

**Annex 3. Details of workshop participants**

| **Participant  code number** | **Country representation** | **Workshop** | **Type of economy (World Bank)*** |
| --- | --- | --- | --- |
| 1 | Argentina | Spanish | UMI |
| 2 | Australia | English 2 | HIC |
| 3 | Australia | English 2 | HIC |
| 4 | Benin | French | LIC |
| 5 | Brazil | English 1 | UMI |
| 6 | Brazil | Spanish | UMI |
| 7 | Burkina Faso | French | LIC |
| 8 | Cameroon | French | LIC |
| 9 | Chile | Spanish | HIC |
| 10 | China | English 2 | HMI |
| 11 | China | English 2 | HMI |
| 12 | Croatia | English 1 | HIC |
| 13 | DRC | French | LIC |
| 14 | Ethiopia | English 1 | LIC |
| 15 | Germany | English 1 | HIC |
| 16 | Guatemala | Spanish | UMI |
| 17 | India | English 2 | LMI |
| 18 | Indonesia | English 2 | LMI |
| 19 | Iran | English 1 | LMI |
| 20 | Kenya | English 1 | LMI |
| 21 | Kenya | English 1 | LMI |
| 22 | Kenya, Uganda, UK | English 1 | LMI, LIC, HIC |
| 23 | Malawi | English 1 | LIC |
| 24 | Pakistan | English 2 | LMI |
| 25 | Peru | Spanish | UMI |
| 26 | Philippines | English 2 | LMI |
| 27 | Spain | Spanish | HIC |
| 28 | Uruguay | Spanish | HIC |
| 29 | US | English 1 | HIC |
| 30 | Viet Nam | English 2 | LMI |
| 31 | Viet Nam | English 2 | LMI |

HIC: High-income country, HMI: Higher middle-income country, LIC: Low-income country,

LMI: Lower middle-income country, UMI: Upper middle-income country

* According to World Bank <https://datahelpdesk.worldbank.org/knowledgebase/articles/906519-world-bank-country-and-lending-groups>
