# Supplementary material for: Engaging women to set the research agenda for assisted vaginal birth
Source: Health Expect. 2024 Jun 14;27(3):e14054. doi: 10.1111/hex.14054 (PMC11178515; doi:10.1111/hex.14054)
Supplement: Supplementary file 4 — Annex 4: Importance and priority of research questions per geographic region. [file HEX-27-e14054-s003.docx]

**Annex 4. Importance and priority of research questions on AVB according to geographical region of participants**

|  | **Importance** | | | | |  | **Priority** | | | | |
| --- | --- | --- | --- | --- | --- | --- | --- | --- | --- | --- | --- |
| **ENGLISH 1** (Brazil, Croatia, Ethiopia, Germany, Iran, Kenya, Malawi, Uganda, US) | Not  important | Low | Moderate | Important | Very  important |  | Very  Low | Low | Moderate | High | Very  high |
| Women´s and communities' views |  |  |  |  |  |  |  |  |  |  |  |
| What knowledge exists among women and communities in LMIC about mode of birth in general? And about AVB? What are their attitudes towards both? |  | 1 | 1 | 1 | 5 |  | 1 |  | 1 | 1 | 5 |
| What barriers do women and communities from LMIC face for AVB? What factors facilitate it? Are there any behavioral or cultural barriers? If so, what are they? |  |  | 1 | 3 | 3 |  |  |  | 2 | 2 | 4 |
| Is there information about AVB in LMIC mass and social media channels? If so, is it accurate and correct? Is it understandable? | 2 |  |  | 4 | 2 |  |  | 1 | 2 | 3 | 1 |
| How does the information available in mass and social media channels impact women’s and communities’ knowledge and attitudes towards AVB in LMIC? |  |  | 3 | 4 | 1 |  |  |  | 3 | 3 | 1 |
| Which channels (TV, social media, radio…) are most effective to inform on AVB? In case of illiteracy, what kind of format (dance, songs, videos…) would be most effective? What communication methods can be used in settings where access to internet and social media is limited? |  |  | 3 | 2 | 2 |  | 1 | 1 | 3 | 1 | 2 |
| Training and clinical aspects |  |  |  |  |  |  |  |  |  |  |  |
| What is the impact of including information and training on AVB courses on its use and outcomes? (e.g., information on maternal and fetal physiology, mechanisms of labor/birth…) |  |  | 1 | 3 | 3 |  |  | 1 |  | 4 | 3 |
| What are the essential elements for effective AVB training? What are essential elements for effective support and supervision? Does it change in different contexts? |  |  | 2 | 1 | 4 |  |  |  | 1 | 3 | 4 |
| Are remote e-learning and mobile technologies acceptable methods to gain and maintain expertise, and to increase AVB use? If so, how? |  | 1 | 2 | 4 |  |  |  | 1 | 4 | 2 | 1 |
| Would a structured international training exchange program be helpful in facilitating and accelerating AVB skills between countries/regions? (Would only be useful if the structure in the receiving setting is in place to allow for AVB) |  |  | 3 | 2 | 2 |  |  |  | 3 | 2 | 3 |
| What is the impact of lack of analgesia on AVB outcomes and views? |  | 1 |  | 3 | 3 |  |  | 1 | 1 | 3 | 3 |
| What are the optimal analgesia methods for AVB? What is the acceptability, outcomes, and resource use associated with the optimal method? |  | 1 |  | 2 | 4 |  |  | 1 |  | 2 | 4 |
| Discovery research: develop and test better and simpler local analgesia methods for AVB that are instrument and setting appropriate. |  |  | 2 | 4 | 2 |  |  | 1 | 1 | 4 | 1 |
| Discovery research: identify new ways/instruments to conduct AVB |  | 1 | 2 | 4 | 1 |  | 1 | 1 | 2 | 3 |  |
| High quality studies comparing outcomes of AVB versus outcomes of a second stage caesarean section so that healthcare professionals and policy makers are better informed |  |  | 1 | 3 | 3 |  |  | 1 |  | 2 | 5 |
| Develop a core outcome set for AVB studies including short- and long-term (5 years or later) maternal and neonatal outcomes that matter to relevant stakeholders (including women) and how to measure these outcomes |  |  | 2 | 2 | 4 |  |  |  | 2 | 2 | 3 |
| Implementation |  |  |  |  |  |  |  |  |  |  |  |
| What are the organizational, cultural, supply and human resources barriers that affect the introduction of AVB techniques in LMIC? What factors facilitate it? |  |  | 1 | 2 | 4 |  |  |  | 1 | 2 | 5 |
| Information and methods on how to organize local maternity services so that every woman who needs it has timely access to a high quality AVB, including pain relief and support |  |  |  | 3 | 4 |  |  |  | 1 | 1 | 6 |
| Economic evaluations for policy-makers and organizations |  |  | 1 | 3 | 4 |  |  |  | 1 | 2 | 4 |
| Sustainability |  |  |  |  |  |  |  |  |  |  |  |
| Where to access AVB? How to facilitate/support AVB in primary healthcare centers? |  |  |  | 2 | 5 |  |  |  |  | 2 | 6 |
| Explore and expand the role of midwives. For example: midwife-led models, or midwife-led birth centers embedded within hospitals which provide comprehensive emergency obstetric and newborn care. Research to assess the feasibility, acceptability and impact of these models in AVB outcomes and outcomes in general. (Depending on regulatory and legal restrictions)  (Must conduct prior research) |  |  | 2 | 1 | 4 |  |  | 1 | 1 | 1 | 5 |
| How to ensure funding and appropriate mechanisms to acquire, replace, and maintain essential equipment including instruments needed for AVB. |  |  | 2 | 2 | 3 |  |  |  | 2 | 2 | 4 |
| What is the impact of Champions*? How to make use of local Champions. If no local Champions are available, how to support international experts in a sustainable and effective manner? What do Champions need? |  | 1 | 2 | 1 | 4 |  |  |  | 3 | 1 | 3 |
| How to ensure appropriate ongoing support to trainees, especially when re-introducing AVB |  |  | 1 | 3 | 4 |  |  | 1 |  | 2 | 4 |
| Explore the impact of regular local/regional audit and feedback (on AVB rates and core maternal and perinatal outcomes) on sustainability of AVB use |  |  | 3 | 1 | 4 |  |  | 1 | 1 | 2 | 3 |
| Explore the long-term impact of including AVB training in the formal curricula of medical schools, residency programs, nursing, and midwifery schools and in-service education programs |  |  | 1 | 4 | 3 |  |  | 1 | 1 | 3 | 2 |
| How to engage policy makers and professional associations more effectively? What strategies are better?. How to engage policy makers to encourage evidence-based practices more effectively. |  |  |  | 3 | 4 |  |  |  | 1 | 2 | 5 |

|  | **Importance** | | | | |  | **Priority** | | | | |
| --- | --- | --- | --- | --- | --- | --- | --- | --- | --- | --- | --- |
| **ENGLISH 2** (Australia, China, India, Indonesia, Pakistan, Philippines, Viet Nam) | Not  important | Low | Moderate | Important | Very  important |  | Very  Low | Low | Moderate | High | Very  high |
| Women and communities' views |  |  |  |  |  |  |  |  |  |  |  |
| What knowledge exists among women and communities in LMIC about mode of birth in general? And about AVB? What are their attitudes towards both? | 1 |  |  | 1 | 3 |  |  |  | 1 | 1 | 2 |
| What barriers do women and communities from LMIC face for AVB? What factors facilitate it? Are there any behavioral or cultural barriers? If so, what are they? |  |  | 2 |  | 2 |  |  |  | 1 | 3 |  |
| Is there information about AVB in LMIC mass and social media channels? If so, is it accurate and correct? Is it understandable? |  |  | 2 | 1 | 1 |  |  | 1 | 1 | 1 | 1 |
| How does the information available in mass and social media channels impact women’s and communities’ knowledge and attitudes towards AVB in LMIC? |  |  | 2 | 1 | 1 |  |  | 1 | 1 | 1 | 1 |
| Which channels (TV, social media, radio…) are most effective to inform on AVB? In case of illiteracy, what kind of format (dance, songs, videos…) would be most effective? What communication methods can be used in settings where access to internet and social media is limited? |  |  |  | 1 | 3 |  |  |  |  | 1 | 3 |
| Training and clinical aspects |  |  |  |  |  |  |  |  |  |  |  |
| What is the impact of including information and training on AVB courses on its use and outcomes? (e.g., information on maternal and fetal physiology, mechanisms of labor/birth…) |  |  | 1 | 2 | 1 |  |  |  | 1 | 2 | 1 |
| What are the essential elements for effective AVB training? What are essential elements for effective support and supervision? Does it change in different contexts? |  |  | 1 | 1 | 2 |  |  |  | 1 |  | 3 |
| Are remote e-learning and mobile technologies acceptable methods to gain and maintain expertise, and to increase AVB use? If so, how? |  | 1 | 2 | 1 |  |  | 1 | 1 | 2 |  |  |
| Would a structured international training exchange program be helpful in facilitating and accelerating AVB skills between countries/regions? (Would only be useful if the structure in the receiving setting is in place to allow for AVB) |  | 1 | 3 |  |  |  |  | 1 | 3 |  |  |
| What is the impact of lack of analgesia on AVB outcomes and views? |  |  | 1 | 1 | 2 |  |  |  | 2 | 1 | 1 |
| What are the optimal analgesia methods for AVB? What is the acceptability, outcomes, and resource use associated with the optimal method? |  |  |  | 2 | 2 |  |  |  |  | 2 | 2 |
| Discovery research: develop and test better and simpler local analgesia methods for AVB that are instrument and setting appropriate. |  |  | 1 | 2 | 1 |  |  |  |  | 2 | 2 |
| Discovery research: identify new ways/instruments to conduct AVB |  | 1 |  | 1 | 2 |  |  | 1 |  | 1 | 2 |
| High quality studies comparing outcomes of AVB versus outcomes of a second stage caesarean section so that healthcare professionals and policy makers are better informed |  |  | 2 |  | 2 |  |  | 2 |  |  | 2 |
| Develop a core outcome set for AVB studies including short- and long-term (5 years or later) maternal and neonatal outcomes that matter to relevant stakeholders (including women) and how to measure these outcomes |  |  | 1 | 1 | 2 |  |  |  | 1 | 1 | 2 |
| Implementation |  |  |  |  |  |  |  |  |  |  |  |
| What are the organizational, cultural, supply and human resources barriers that affect the introduction of AVB techniques in LMIC? What factors facilitate it? |  |  | 1 | 1 | 2 |  |  |  | 1 |  | 3 |
| Information and methods on how to organize local maternity services so that every woman who needs it has timely access to a high quality AVB, including pain relief and support |  | 1 |  | 2 | 1 |  |  | 1 |  | 2 | 1 |
| Economic evaluations for policy-makers and organizations |  | 2 | 1 |  | 1 |  |  | 3 |  |  | 1 |
| Sustainability |  |  |  |  |  |  |  |  |  |  |  |
| Where to access AVB? How to facilitate/support AVB in primary healthcare centers? |  |  | 2 | 1 | 1 |  |  | 1 | 1 | 1 | 1 |
| Explore and expand the role of midwives. For example: midwife-led models, or midwife-led birth centers embedded within hospitals which provide comprehensive emergency obstetric and newborn care. Research to assess the feasibility, acceptability and impact of these models in AVB outcomes and outcomes in general. (Depending on regulatory and legal restrictions) (Must conduct prior research) |  |  |  | 2 | 2 |  |  |  |  | 2 | 2 |
| How to ensure funding and appropriate mechanisms to acquire, replace, and maintain essential equipment including instruments needed for AVB. |  |  | 1 | 2 | 1 |  |  |  | 1 | 3 |  |
| What is the impact of Champions*? How to make use of local Champions. If no local Champions are available, how to support international experts in a sustainable and effective manner? What do Champions need? |  | 2 | 1 | 1 |  |  | 1 | 1 | 1 | 1 |  |
| How to ensure appropriate ongoing support to trainees, especially when re-introducing AVB |  |  | 1 |  | 3 |  |  | 1 |  |  | 3 |
| Explore the impact of regular local/regional audit and feedback (on AVB rates and core maternal and perinatal outcomes) on sustainability of AVB use |  |  | 1 | 1 | 2 |  |  |  |  | 3 | 1 |
| Explore the long-term impact of including AVB training in the formal curricula of medical schools, residency programs, nursing, and midwifery schools and in-service education programs |  |  | 1 | 1 | 2 |  |  |  | 1 | 1 | 2 |
| How to engage policy makers and professional associations more effectively? What strategies are better? How to engage policy makers to encourage evidence-based practices more effectively. |  | 1 | 1 | 1 | 1 |  |  | 2 | 1 |  | 1 |

|  | **Importance** | | | | |  | **Priority** | | | | |
| --- | --- | --- | --- | --- | --- | --- | --- | --- | --- | --- | --- |
| **SPANISH** (Argentina, Brazil, Chile, Guatemala, Peru, Spain, Uruguay) | Not  important | Low | Moderate | Important | Very  important |  | Very  Low | Low | Moderate | High | Very  high |
| Women and communities' views |  |  |  |  |  |  |  |  |  |  |  |
| What knowledge exists among women and communities in LMIC about mode of birth in general? And about AVB? What are their attitudes towards both? |  | 1 | 1 |  | 4 |  | 1 |  |  | 1 | 3 |
| What barriers do women and communities from LMIC face for AVB? What factors facilitate it? Are there any behavioral or cultural barriers? If so, what are they? |  | 1 | 1 |  | 4 |  |  |  |  | 2 | 3 |
| Is there information about AVB in LMIC mass and social media channels? If so, is it accurate and correct? Is it understandable? | 1 | 2 |  | 1 | 2 |  | 2 | 1 |  | 1 | 2 |
| How does the information available in mass and social media channels impact women’s and communities’ knowledge and attitudes towards AVB in LMIC? |  | 1 | 1 | 2 | 2 |  |  | 1 | 1 | 2 | 2 |
| Which channels (TV, social media, radio…) are most effective to inform on AVB? In case of illiteracy, what kind of format (dance, songs, videos…) would be most effective? What communication methods can be used in settings where access to internet and social media is limited? |  |  |  | 2 | 3 |  |  |  |  | 3 | 2 |
| Training and clinical aspects |  |  |  |  |  |  |  |  |  |  |  |
| What is the impact of including information and training on AVB courses on its use and outcomes? (e.g., information on maternal and fetal physiology, mechanisms of labor/birth…) |  |  |  |  | 5 |  |  |  |  | 2 | 3 |
| What are the essential elements for effective AVB training? What are essential elements for effective support and supervision? Does it change in different contexts? |  |  |  | 3 | 2 |  |  |  | 1 | 2 | 2 |
| Are remote e-learning and mobile technologies acceptable methods to gain and maintain expertise, and to increase AVB use? If so, how? |  | 1 | 1 | 1 | 3 |  |  |  | 3 | 1 | 2 |
| Would a structured international training exchange program be helpful in facilitating and accelerating AVB skills between countries/regions? (Would only be useful if the structure in the receiving setting is in place to allow for AVB) |  |  | 2 | 3 | 1 |  |  |  | 3 | 2 | 1 |
| What is the impact of lack of analgesia on AVB outcomes and views? |  | 1 | 1 | 2 | 2 |  |  | 1 | 1 | 2 | 2 |
| What are the optimal analgesia methods for AVB? What is the acceptability, outcomes, and resource use associated with the optimal method? |  | 1 | 2 | 1 | 2 |  |  | 1 | 2 | 1 | 2 |
| Discovery research: develop and test better and simpler local analgesia methods for AVB that are instrument and setting appropriate. |  |  | 2 | 2 | 2 |  |  |  | 2 | 2 | 2 |
| Discovery research: identify new ways/instruments to conduct AVB |  | 1 | 2 | 3 | 1 |  |  | 1 | 3 | 2 | 1 |
| High quality studies comparing outcomes of AVB versus outcomes of a second stage caesarean section so that healthcare professionals and policy makers are better informed |  |  | 1 | 3 | 2 |  |  |  | 2 | 2 | 2 |
| Develop a core outcome set for AVB studies including short- and long-term (5 years or later) maternal and neonatal outcomes that matter to relevant stakeholders (including women) and how to measure these outcomes |  |  |  | 3 | 3 |  |  |  | 1 | 2 | 3 |
| Implementation |  |  |  |  |  |  |  |  |  |  |  |
| What are the organizational, cultural, supply and human resources barriers that affect the introduction of AVB techniques in LMIC? What factors facilitate it? |  |  |  | 4 | 2 |  |  |  | 1 | 3 | 2 |
| Information and methods on how to organize local maternity services so that every woman who needs it has timely access to a high quality AVB, including pain relief and support |  | 1 |  | 1 | 4 |  |  | 1 | 1 |  | 4 |
| Economic evaluations for policy-makers and organizations |  |  | 3 |  | 3 |  |  |  | 2 | 1 | 2 |
| Sustainability |  |  |  |  |  |  |  |  |  |  |  |
| Where to access AVB? How to facilitate/support AVB in primary healthcare centers? |  |  | 2 | 2 | 2 |  |  |  | 2 | 2 | 2 |
| Explore and expand the role of midwives. For example: midwife-led models, or midwife-led birth centers embedded within hospitals which provide comprehensive emergency obstetric and newborn care. Research to assess the feasibility, acceptability and impact of these models in AVB outcomes and outcomes in general. (Depending on regulatory and legal restrictions)  (Must conduct prior research) | 1 | 1 |  |  | 4 |  | 2 |  |  |  | 4 |
| How to ensure funding and appropriate mechanisms to acquire, replace, and maintain essential equipment including instruments needed for AVB. |  |  | 2 | 3 | 2 |  |  |  | 3 | 2 | 2 |
| What is the impact of Champions*? How to make use of local Champions. If no local Champions are available, how to support international experts in a sustainable and effective manner? What do Champions need? | 2 | 2 |  | 2 | 1 |  | 2 | 2 |  | 2 | 1 |
| How to ensure appropriate ongoing support to trainees, especially when re-introducing AVB |  |  | 1 | 4 | 2 |  |  |  | 2 | 3 | 2 |
| Explore the impact of regular local/regional audit and feedback (on AVB rates and core maternal and perinatal outcomes) on sustainability of AVB use |  | 1 | 1 | 1 | 3 |  |  | 1 | 1 | 1 | 3 |
| Explore the long-term impact of including AVB training in the formal curricula of medical schools, residency programs, nursing, and midwifery schools and in-service education programs |  |  | 1 | 4 | 2 |  |  |  | 1 | 4 | 2 |
| How to engage policy makers and professional associations more effectively? What strategies are better?. How to engage policy makers to encourage evidence-based practices more effectively. |  | 1 | 1 | 2 | 2 |  |  | 1 | 1 | 2 | 2 |

|  | **Importance** | | | | |  | **Priority** | | | | |
| --- | --- | --- | --- | --- | --- | --- | --- | --- | --- | --- | --- |
| **FRENCH** (Benin, Burkina Faso, Cameroon, DRC) | Not  important | Low | Moderate | Important | Very  important |  | Very  Low | Low | Moderate | High | Very  high |
| Women and communities' views |  |  |  |  |  |  |  |  |  |  |  |
| What knowledge exists among women and communities in LMIC about mode of birth in general? And about AVB? What are their attitudes towards both? |  |  |  | 2 | 1 |  |  |  |  | 1 | 2 |
| What barriers do women and communities from LMIC face for AVB? What factors facilitate it? Are there any behavioral or cultural barriers? If so, what are they? |  |  |  | 1 | 2 |  |  |  | 1 |  | 2 |
| Is there information about AVB in LMIC mass and social media channels? If so, is it accurate and correct? Is it understandable? | 1 |  | 1 | 1 |  |  | 1 |  |  |  | 2 |
| How does the information available in mass and social media channels impact women’s and communities’ knowledge and attitudes towards AVB in LMIC? |  |  |  | 2 | 1 |  |  |  | 1 | 1 | 1 |
| Which channels (TV, social media, radio…) are most effective to inform on AVB? In case of illiteracy, what kind of format (dance, songs, videos…) would be most effective? What communication methods can be used in settings where access to internet and social media is limited? |  |  |  | 1 | 2 |  |  |  |  | 1 | 2 |
| Training and clinical aspects |  |  |  |  |  |  |  |  |  |  |  |
| What is the impact of including information and training on AVB courses on its use and outcomes? (e.g., information on maternal and fetal physiology, mechanisms of labor/birth…) |  |  | 1 |  | 2 |  |  |  |  | 1 | 2 |
| What are the essential elements for effective AVB training? What are essential elements for effective support and supervision? Does it change in different contexts? |  |  |  | 2 | 1 |  |  |  |  | 1 | 2 |
| Are remote e-learning and mobile technologies acceptable methods to gain and maintain expertise, and to increase AVB use? If so, how? |  | 1 |  | 1 | 1 |  |  |  | 2 |  | 1 |
| Would a structured international training exchange programs be helpful in facilitating and accelerating AVB skills between countries/regions? (Would only be useful if the structure in the receiving setting is in place to allow for AVB) |  |  |  | 1 | 2 |  |  |  |  | 1 | 2 |
| What is the impact of lack of analgesia on AVB outcomes and views? |  |  |  | 3 |  |  |  |  | 1 |  | 2 |
| What are the optimal analgesia methods for AVB? What is the acceptability, outcomes, and resource use associated with the optimal method? |  |  | 1 | 2 |  |  |  | 1 |  |  | 2 |
| Discovery research: develop and test better and simpler local analgesia methods for AVB that are instrument and setting appropriate. |  |  |  | 2 | 1 |  |  |  |  | 2 | 1 |
| Discovery research: identify new ways/instruments to conduct AVB |  |  |  | 1 | 2 |  |  |  |  | 3 |  |
| High quality studies comparing outcomes of AVB versus outcomes of a second stage caesarean section so that healthcare professionals and policy makers are better informed |  |  |  | 1 | 2 |  |  |  |  |  | 3 |
| Develop a core outcome set for AVB studies including short- and long-term (5 years or later) maternal and neonatal outcomes that matter to relevant stakeholders (including women) and how to measure these outcomes |  |  | 1 |  | 2 |  |  |  |  | 1 | 2 |
| Implementation |  |  |  |  |  |  |  |  |  |  |  |
| What are the organizational, cultural, supply and human resources barriers that affect the introduction of AVB techniques in LMIC? What factors facilitate it? |  |  |  | 2 | 1 |  |  |  |  | 1 | 2 |
| Information and methods on how to organize local maternity services so that every woman who needs it has timely access to a high quality AVB, including pain relief and support |  |  |  |  | 3 |  |  |  |  |  | 3 |
| Economic evaluations for policy-makers and organizations |  |  | 1 |  | 2 |  |  |  |  |  | 3 |
| Sustainability |  |  |  |  |  |  |  |  |  |  |  |
| Where to access AVB? How to facilitate/support AVB in primary healthcare centers? |  |  |  | 2 | 1 |  |  |  |  |  | 3 |
| Explore and expand the role of midwives. For example: midwife-led models, or midwife-led birth centers embedded within hospitals which provide comprehensive emergency obstetric and newborn care. Research to assess the feasibility, acceptability and impact of these models in AVB outcomes and outcomes in general. (Depending on regulatory and legal restrictions)  (Must conduct prior research) |  |  |  | 1 | 2 |  |  |  |  |  | 3 |
| How to ensure funding and appropriate mechanisms to acquire, replace, and maintain essential equipment including instruments needed for AVB. |  |  |  | 1 | 2 |  |  |  |  |  | 3 |
| What is the impact of Champions*? How to make use of local Champions. If no local Champions are available, how to support international experts in a sustainable and effective manner? What do Champions need? |  |  |  |  | 3 |  |  |  |  |  | 3 |
| How to ensure appropriate ongoing support to trainees, especially when re-introducing AVB |  |  |  | 1 | 2 |  |  |  |  | 3 |  |
| Explore the impact of regular local/regional audit and feedback (on AVB rates and core maternal and perinatal outcomes) on sustainability of AVB use |  |  |  | 1 | 2 |  |  |  |  | 1 | 2 |
| Explore the long-term impact of including AVB training in the formal curricula of medical schools, residency programs, nursing, and midwifery schools and in-service education programs |  |  |  |  | 3 |  |  |  |  |  | 3 |
| How to engage policy makers and professional associations more effectively? What strategies are better?. How to engage policy makers to encourage evidence-based practices more effectively. |  |  |  |  | 3 |  |  |  |  |  | 3 |

Numbers indicate N of participants who clicked on each answer. AVB: Assisted vaginal birth, CS: Cesarean section, , HCP: Health care provider
