# Supplementary material for: Engaging women to set the research agenda for assisted vaginal birth
Source: Health Expect. 2024 Jun 14;27(3):e14054. doi: 10.1111/hex.14054 (PMC11178515; doi:10.1111/hex.14054)
Supplement: Supplementary file 5 — Annex 5. Views and comments on research categories per geographic region. [file HEX-27-e14054-s004.docx]

**Annex 5. Views and comments on research categories per geographic region**

| **Women´s and Communities´ views on AVB** | | | | | |
| --- | --- | --- | --- | --- | --- |
| **Workshop group** | **Spanish*** | **French*** | **English 1*** | **English 2*** | **Total** |
| Safety concerns (fears of damage to baby and maternal physical and emotional health) need to be addressed. | x | x | x | x | 4 |
| Women lack knowledge that AVB is an option when VD is not possible. This lack of knowledge leads them to see CS as the only alternative. This must to be addressed. |  | x | x | x | 3 |
| It is key to understand how AVB is framed by women, and understand how and where women obtain, process and are influenced by information about AVB. |  | x | x | x | 3 |
| Research is needed on how to change the view of some communities/societies where birth is seen as an illness instead of a physiological process. | x |  | x | x | 3 |
| Education is key to avoid misconceptions and harmful beliefs and for decision-making |  |  | x | x | 2 |
| It is important to consider the influence of communities to which women belong | x |  |  | x | 2 |
| It is important not only to assess women´s and communities´ views and opinions about AVB but also to study how much the opinions/views of women and communities about AVB are taken into consideration by researchers, policy makers and other relevant actors. | x |  |  |  | 1 |
| It will be challenging to get women´s views about AVB in settings where this intervention is almost inexistent | x |  |  |  | 1 |
| Investigate how to use sensibilization strategies to involve policy makers and HCP to invest efforts into providing information about AVB to the general population |  | x |  |  | 1 |
| Research on the sources of influence and the role of decision-aids |  |  |  | x | 1 |
| Total | 5 | 4 | 5 | 7 | 21 |

| **AVB Training and Clinical Aspects** | | | | | |
| --- | --- | --- | --- | --- | --- |
| **Workshop group** | **Spanish*** | **French*** | **English 1*** | **English 2*** | **Total** |
| It is important to train HCPs about non-technical skill, such as communication skills and how to treat women with empathy and respect and how to see women not as objects but as subjects capable of participating in decision making | x | x | x | x | 4 |
| Research on how to train and sustain skills and knowledge at point of care is fundamental | x | x | x | x | 4 |
| It is important to teach/train HCPs (including midwives) to view labor and childbirth as healthy physiological processes that should be respected and how to best support women physically and emotionally during these processes | x | x | x | x | 4 |
| It will be necessary to renovate buildings and delivery rooms to offer AVB(clinical practice depends on structural changes) |  | x | x |  | 2 |
| In remote settings (especially in Africa) on-line training and exchange of clinical information between HCPs is important |  | x |  | x | 2 |
| In some settings (Benin), some older midwives may not have the technological skills to participate in on-line training |  | x |  |  | 1 |
| It is important to motivate and train HCPs who do not want to learn how to do AVBs |  | x |  |  | 1 |
| Explore possibility of hybrid clinician training models facilitated by midwives so that the role of the midwife is integrated into the clinician’s practice as well. |  |  |  | x | 1 |
| Ensure that companionship is allowed in the moment that the women is most vulnerable | x |  |  |  | 1 |
| Total | 4 | 7 | 4 | 5 | 20 |

| **Implementation of AVB** | | | | | |
| --- | --- | --- | --- | --- | --- |
| **Workshop group** | **Spanish*** | **French*** | **English 1*** | **English 2*** | **Total** |
| Financial resources and the supply of instruments are essential for implementation | x | x | x |  | 3 |
| Active engagement and participation of government and policy makers is essential for change. | x |  | x | x | 3 |
| It is very difficult to implement what has been taught (knowing - doing gap) |  | x |  | x | 2 |
| It is important to think about how to manage political and financial barriers, and insurance limitations (if CSs are more profitable for the system, it will be difficult to change) |  |  | x | x | 2 |
| Health systems are perceived to meet the needs of providers before those of women. Strategies need to address the concerns of both |  |  |  | x | 1 |
| Human rights must be ensured during implementation | x |  |  |  | 1 |
| Managerial capacity is essential for implementation to work | x |  |  |  | 1 |
| Sanitation and hygiene need to be ensured |  |  | x |  | 1 |
| Total | 3 | 2 | 4 | 4 | 14 |

| **Sustainability of AVB** | | | | | |
| --- | --- | --- | --- | --- | --- |
| **Topic** | **Spanish*** | **French*** | **English 1*** | **English 2*** | **Total** |
| Information must be provided to all actors involved: women, clinicians, midwives | x | x | x | x | 4 |
| Task-shifting: it is important that midwives be allowed to do AVB to ensure the sustainability of AVB, especially in rural and remote areas | x |  | x | x | 3 |
| Midwives need to be given more power, skill sets and knowledge. Their competences and abilities must be recognized |  |  | x | x | 2 |
| In some countries, "AVB champions" may have a negative impact because associated incentives to perform AVB may disregard and run over women´s opinions and rights. Therefore, it is important to investigate possible negative effects of "champions" on AVB use/acceptability. | x |  |  |  | 1 |
| It is essential to have financial and managerial strategies to ensure the continued availability of functioning instruments and other necessary equipment for AVB |  | x |  |  | 1 |
| Clear guidelines on indications for AVB and CS are needed |  |  | x |  | 1 |
| Total | 3 | 2 | 4 | 3 | 12 |

Number of times mentioned by participants

AVB: Assisted vaginal birth, CS: Cesarean section, , HCP: Health care provider, VD: Vaginal delivery

*Nationality of women´s representatives in each workshop:

ENGLISH 1: Brazil, Croatia, Ethiopia, Germany, Iran, Kenya, Malawi, Uganda, US

ENGLISH 2: Australia, China, India, Indonesia, Pakistan, Philippines, Viet Nam

FRENCH: Benin, Burkina Faso, Cameroon, DRC

SPANISH: Argentina, Brazil, Chile, Guatemala, Peru, Spain, Uruguay
