# Supplementary material for: Engaging women to set the research agenda for assisted vaginal birth
Source: Health Expect. 2024 Jun 14;27(3):e14054. doi: 10.1111/hex.14054 (PMC11178515; doi:10.1111/hex.14054)
Supplement: Supplementary file 6 — Annex 6. Concerns of women about AVB per geographic region. [file HEX-27-e14054-s002.docx]

**Annex 6. Concerns of women about AVB according to geographic region**

| **Topic** | **Spanish*** | **French*** | **English 1*** | **English 2*** | **Total** |
| --- | --- | --- | --- | --- | --- |
| ***Safety*** | | | | | |
| Safety of AVB for baby | x | x | x | x | 4 |
| Safety of AVB for women (physical and emotional/mental health) | x |  | x | x | 3 |
| Unavailability of treatment, follow-up and support in case of adverse maternal or neonatal outcomes | x |  |  | x | 2 |
| ***Healthcare providers skills and health systems*** | | | | | |
| Fear that HCPs are not competent/skilled to do AVB | x | x |  | x | 3 |
| Maternal risks due to lack of hygiene of instruments and rooms where AVB occur |  | x |  |  | 1 |
| The fear that women have of midwives. |  | x |  |  | 1 |
| Disrespect of HCPs for physiology of labour and birth, non-humanized care leading to maternal trauma | x |  | x | x | 3 |
| Excess use of unnecessary AVBs as part of the obstetric violence scenario or effort to decrease CS rates | x |  |  |  | 1 |
| Social inequality in access to good quality health care by most vulnerable/ poorest pregnant women | x |  |  |  | 1 |
| ***Communication and information*** | | | | | |
| Poor communication between health care providers and women |  | x | x | x | 3 |
| Pressure put on women to sign AVB informed consent without adequate information | x | x |  | x | 3 |
| Women´s insufficient information about AVB which leads to fear |  | x | x | x | 3 |
| Total | 8 | 7 | 5 | 8 | 28 |

Number of times mentioned by participants

AVB: Assisted vaginal birth, CS: Cesarean section, , HCP: Health care provider

*Nationality of women´s representatives in each workshop:

ENGLISH 1: Brazil, Croatia, Ethiopia, Germany, Iran, Kenya, Malawi, Uganda, US

ENGLISH 2: Australia, China, India, Indonesia, Pakistan, Philippines, Viet Nam

FRENCH: Benin, Burkina Faso, Cameroon, DRC

SPANISH: Argentina, Brazil, Chile, Guatemala, Peru, Spain, Uruguay
